# PML-nuclear bodies decrease with age and their stress response is impaired in aged individuals

**DOI:** 10.1186/1471-2318-14-42

**Published:** 2014-04-02

**Authors:** Barbara Wenger, Manuela Schwegler, Maria Brunner, Christoph Daniel, Manfred Schmidt, Rainer Fietkau, Luitpold V Distel

**Affiliations:** 1 Department of Radiation Oncology, University Hospitals and Friedrich-Alexander-University Erlangen-Nürnberg, Universitätsstraße 27, D-91054 Erlangen, Germany; 2 Institute of Pathology, Department of Nephropathology, University Hospitals and Friedrich-Alexander-University Erlangen-Nürnberg, Erlangen, Germany

**Keywords:** Aging, PML Nuclear bodies, γH2AX, Stress response, Senescence

## Abstract

**Background:**

Promyelocytic leukemia nuclear bodies (PML-NBs) have been depicted as structures which are involved in processing cell damages and DNA double-strand break repairs. The study was designed to evaluate differences in patients’ PML-NBs response to stress factors like a cancerous disease and ionizing radiation exposure dependent on age.

**Methods:**

In order to clarify the role of PML-NBs in the aging process, we examined peripheral blood monocytes of 134 cancer patients and 41 healthy individuals between 22 and 92 years of age, both before and after *in vitro* irradiation. Additionally, we analyzed the samples of the cancer patients after *in vivo* irradiation. Cells were immunostained and about 1600 cells per individual were analyzed for the presence of PML- and γH2AX foci.

**Results:**

The number of existing PML-NBs per nucleus declined with age, while the number of γH2AX foci increased with age. There was a non-significant trend that *in vivo* irradiation increased the number of PML-NBs in cells of young study participants, while in older individuals PML-NBs tended to decrease. It can be assumed that PML-NBs decrease in number during the process of aging.

**Conclusion:**

The findings suggest that there is a dysfunctional PML-NBs stress response in aged cells.

## Background

Promyelocytic leukemia protein (PML) is part of a large multiprotein nuclear complex known as promyelocytic leukemia nuclear bodies (PML-NBs), which is associated with the nuclear matrix [1]. Promyelocytic leukemia protein is involved in multiple cellular functions including transcription, chromatin dynamics, oncogenesis, posttranslational modifications and DNA damage response [2,3]. Additionally, PML plays a role in senescence and ageing. PML is upregulated during cellular senescence [4] and induces permanent cell cycle arrest via p53 and retinoblastoma protein regulation [5]. At the same time, PML is a dynamic sensor of DNA damage and cellular stress [6] and it is involved in a later step of the DNA double-strand break (DSB) repair [7]. In response to DNA damage, the number of PML-NBs increases and these PML-NBs alter their subnuclear location [7]. One type of cellular stress is ionizing radiation due to its DNA damaging properties. The number of DSBs identified as γH2AX foci in peripheral blood monocytes is measured in order to assess the stress triggered by irradiating the cells. γH2AX foci are formed at the sites of DSB by the phosphorylation of the H2A histone variant H2AX at its amino acid serine 139 [8]. The ratio of visible γH2AX foci to DSBs is close to 1:1, what makes the γH2AX immunofluorescent staining the most sensitive method available for detecting DSBs in human cells [9]. Individual differences in DSB repair can be obtained from interindividual disparities in the number of γH2AX foci per nucleus after *in vitro* irradiation and the subsequent repair. The highest numbers of double strand breaks per nucleus are visualized 30 minutes post irradiation. The DSB number declines over a period of hours, while the cells are repairing the DNA damage.

We evaluated the difference in patients’ PML response to stress factors like cancer and radiotherapy treatment dependent on age. We investigated the number of PML-NBs in peripheral blood mononuclear cells of healthy individuals and cancer patients of different ages. Pre-existing and via ionizing radiation induced PML-NBs were studied. As a measure of cellular stress, we compared the PML-NBs with the number of DNA double-strand breaks.

## Method

### Patients characteristics

The prospective study included a total of 175 individuals. 66 rectal cancer patients (RC), 68 breast cancer patients (BC) and 41 healthy participants were enrolled. The trial was in compliance with the WMA Declaration of Helsinki - Ethical Principles for Medical Research Involving Human Subjects. All patients and healthy individuals gave their written informed consent. This study was approved by the ethics review committees of the Friedrich-Alexander-Universität Erlangen-Nürnberg (No. 2725). The patients’ blood samples were taken immediately before the first irradiation fraction to yield the pre-existing background rates. After a daily fractionated radiation treatment (5 × 1.8 Gy) and a free interval of three days the second blood sample was taken (*in vivo* irradiation). RC patients were treated with 5-fluoruracil (5-FU) or a combination of 5-FU and oxaliplatin once a week. BC patients received docetaxel, cyclophosphamide, epirubicin or paclitaxel or a combination of these substances. Some BC patients additionally received an antihormonal therapy.

### Blood samples and peripheral blood mononucleated cells separation

The blood samples taken from patients prior to radiochemotherapy (RCT) and the blood samples taken from control group were split into three samples. Two different doses had to be used to irradiate the peripheral blood mononucleated cells, because a dose of 2 Gy would induce after 30 minutes such a high amount of foci so that they could not be separated from each other and counted properly. There would remain only a very low number of foci 24 h after the low dose of 0.5 Gy and therefore the statistics would be poor. Though the first sample was *in vitro* irradiated using 0.5 Gy and afterwards incubated for 30 minutes. This procedure was adopted for the second sample with the difference of using 2 Gy and having a 24 h incubation time. The third sample served as control. After five fractions *in vivo* irradiation and the free interval of three days, the fourth sample was taken. Two identical cover slips were produced from each sample. Briefly, peripheral blood mononucleated cells (PBMC) were isolated from heparinized whole blood samples by Ficoll gradient centrifugation. PBMC were maintained in RPMI 1640 medium supplemented with 1% penicillin-streptomycin and 10% fetal calf serum. PBMC were divided into three samples, two were *in vitro* irradiated and the third sample was used as a control. Afterwards, the blood samples were cytocentrifuged (StatspinCytofuge, Kreatech, Germany) onto specimen. The PBMC were fixed for 30 minutes in methanol and for one minute in acetone before they were washed for 3x10 minutes in a phosphate-buffered saline with foetal calf serum.

### Primary fibroblasts

A 2-mm punch skin biopsy was taken from the forearm of a healthy Caucasian individual. The dermis was cut into small pieces and placed in a small flask where it was covered with F12 medium (Biochrom, Berlin, Germany) containing 20% fetal calf serum. The outgrowing fibroblasts were trypsinized and sub-cultured.

### Antibodies and immunofluorescence analysis

One of the two identical cover slips of each approach was then incubated with the mouse anti-γH2AX antibody (Abcam, Cambridge, UK) and the second cover slip with the rabbit anti-PML antibody (Santa Cruz, CA, USA). Afterwards the cover slips were washed in PBS three times and then incubated with a secondary goat anti-mouse labeled with Alexa 488 fluorescent antibody or goat anti-rabbit labeled with Alexa 594 fluorescent antibody (Molecular Probes, Karlsruhe, Germany). Then, the samples were washed in PBS again three times and mounted by using the Vectashield mounting medium (Vector Laboratories, Peterborough, UK).

Fluorescence labeled blood cells were visualized by a fluorescence-microscope (Axioplan 2, Zeiss, Göttingen, Germany) and image acquisition software (Metafer 4, MetaSystems, Altlußheim, Germany). Digital images of five optical planes separated by a distance of 0.75 μm were recorded and combined to an extended focus image using the maximum intensity algorithm (Metasystems). An area of 2 mm^2^ (630×) was captured automatically. For each of the samples 500 to 1000 cells were identified by using image analysis software (Biomas, Erlangen, Germany). All nuclei were morphologically considered by eye to be properly shaped and cells in cell-division phase were excluded. By using Biomas, the PML foci and the γH2AX foci inside each nucleus were counted [10]. The number of mean residual foci per cell was determined for every individual before irradiation, 30 minutes after a dose of 0.5 Gy and 24 h after a dose of 2 Gy. After the five fractions *in vivo* irradiation and the free interval of three days the mean number of foci per cell was counted again.

### *In vivo* exposed dose

We evaluated two quantities to consider dose aspects: The total deposit energy E_dep_ and the mean dose D_mean_, which is the dose averaged over the whole body of the patient. These quantities are strongly connected by a proportionality relation:

$$D_{\mathrm{mean}}=\frac{E_{\mathrm{dep}}}{m},$$

where m is the mass of the patient.

The total deposit energy E_dep_ is estimated (here) by:

$$E_{\mathrm{dep}}=\sum_{k}\mathrm{pk}\times D_{\mathrm{pres}.}\left(V_{\mathrm{pk}}-V_{\mathrm{pk}+1}\right)\times \rho ,$$

where ρ is the mass density of the patient (ρ was approximately set to 1 kg/dm^3^), D_pres._ is the prescribed dose and V_p_ is the isodose volume with at least a percentage p of the prescribed dose. We used p in the following percentage steps: 0.2, 0.3 0.4, 0.6, 0.8, 0.9 and 0.95. The different volumes Vp are achieved from the dose distribution in the patients given by the treatment planning system (Pinnacle, Philips, Fitchburg, WI, USA).

### Statistical analysis

The independent *t*-test was used to test for statistical differences. With p < 0.05, differences were considered statistically significant. Pearson correlation was used to evaluate a possible correlation between PML-NBs frequency per cell and time after exposure and between age and focus formation. Statistical calculation was performed by using SPSS version 19 (IBM, Ehningen, Germany).

## Results

### Study population

**Figure 1:**
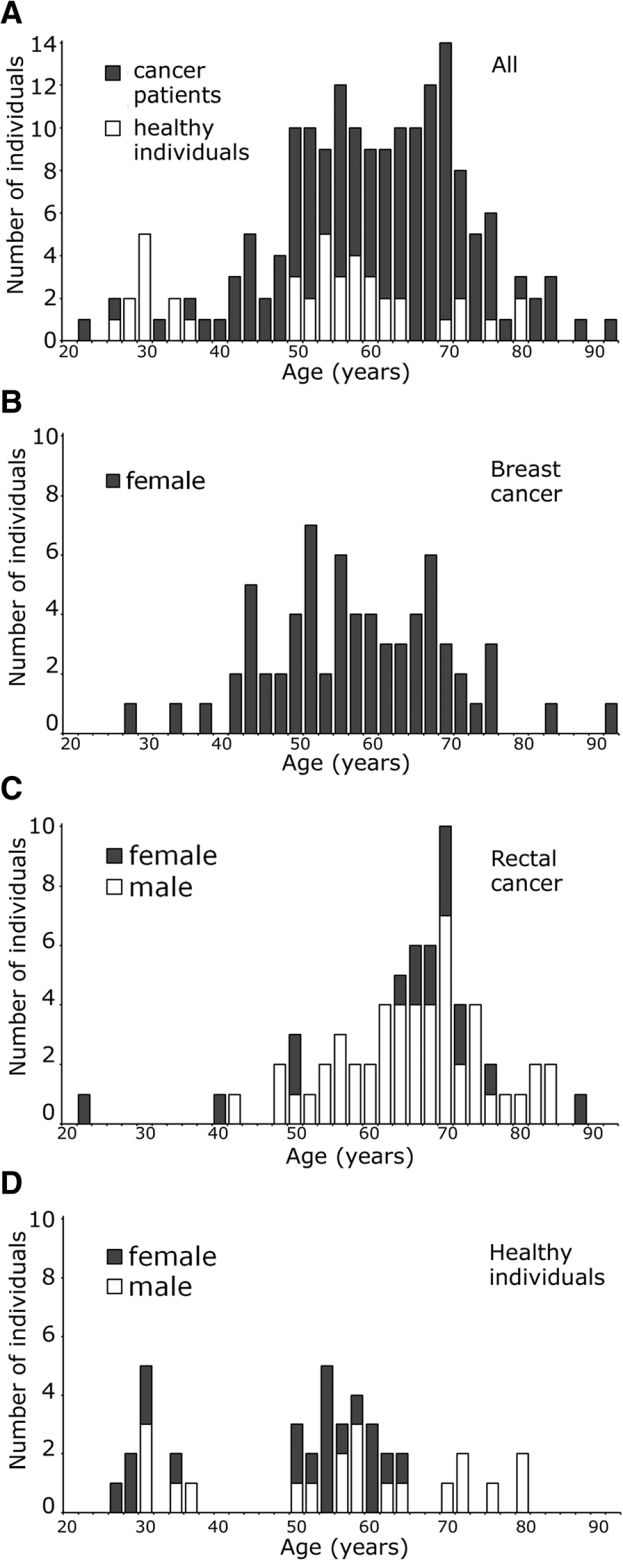
**Study group characteristics. (A)** Distribution by age and gender for the group of patients and healthy individuals altogether, **(B)** for the patient group with breast cancer, **(C)** the patient group with rectal cancer and **(D)** healthy individuals.

Our study group included a total of 175 individuals (Figure 1A). Patients enrolled were between 22 and 92 years of age. Within this group 68 patients were with breast cancer (BC) (Figure 1B), who received adjuvant radiochemotherapy, and 66 patients with rectal cancer (RC) (Figure 1C), who received neoadjuvant radiochemotherapy. The *in vivo* irradiation of RC patients included five days of radiotherapy followed by a free interval of three days (*in vivo* irradiation). The group of patients included 54 males and 80 females. Our healthy group consisted of 41 healthy individuals between 26 and 82 years of age. This group included 20 males and 21 females (Figure 1D). The clinical characteristics of the study group are listed in Table 1.

**Table 1:** Patients’ characteristics

	**175 individuals**				
	**Healthy controls**	**Rectal cancer patients n (%)**		**Breast cancer patients (%)**	
n	41		66		68
Mean age	52.3a (±14.0a)		63.7a (±11.2a)		56.5a (±11.7a)
	-	cT2/cT3/cT4	2/61/3 (3.0/92.4/4.6)	Tis/T1/T2/T3/T4	6/37/23/1/1
	-	cN0/cN+	12/54 (18.2/81.8)	N0/N+	45/23
	-	cM0/cM1	54/12 (81.8/18.2)	M0/M1	65/3
	-	Stage 3/4	58/8 (87.8/12.2)	Dcis/no Dcis	38/30
	-			Mastectomy	9
				Breast-preserving	59

### γH2AX and PML foci in fibroblasts and PBMC

**Figure 2:**
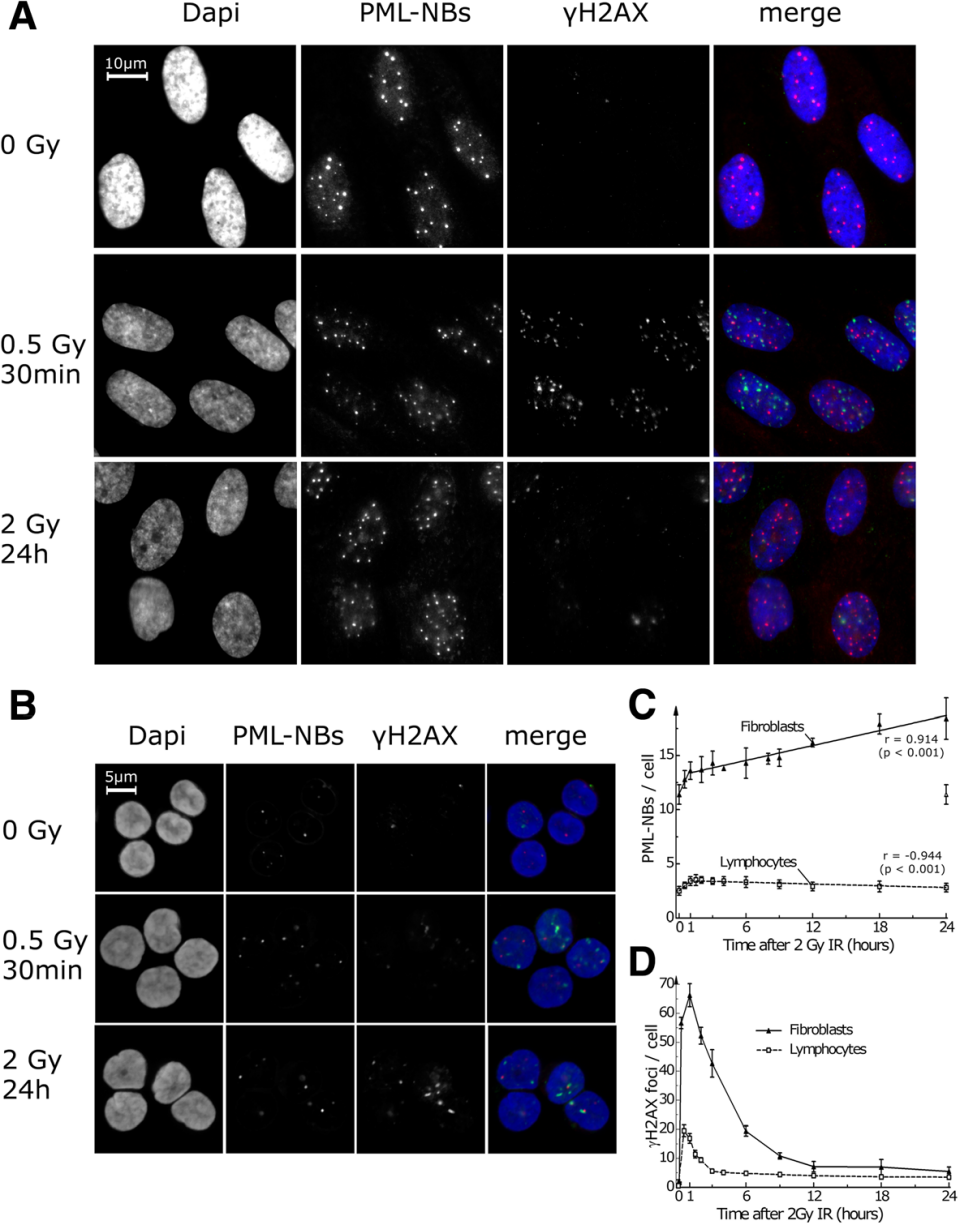
**Time dependence of PML-NBs induction after ionizing radiation.** Fluorescence microscopic images of **(A)** fibroblasts and **(B)** PBMC before and after irradiation by X-rays stained with anti-γH2AX and anti-PML. The number of PML-NBs **(C)** and number of γH2AX foci **(D)** in irradiated cells using 2 Gy were counted at different times after irradiation. The black triangles represent fibroblasts and the white squares PBMC’s. Pearson’s correlation coefficients (r) and the p-values for the linear function are given **(C)**.

In order to investigate whether there are any differences in fibroblast (Figure 2A) and PBMC (Figure 2B) PML and γH2AX focus formation, we irradiated both types of cells and measured the incidence of PML and γH2AX foci. In fibroblasts the PML focus formation increased rapidly until 1 hour after irradiation using 2 Gy and the number of PML foci rose slowly during the next 24 hours. In peripheral blood monocytes the number increased during the first hour and decreased very slowly throughout the following 24 hours (Figure 2C). After irradiating peripheral blood cells using 2 Gy, the count of γH2AX foci formation reached its maximum after 30 minutes and declined over the next hours (Figure 2D). The number of γH2AX foci in fibroblasts reached its maximum 1 hour after irradiation and declined biphasic during the following 12 hours.

### Incidence of PML-NBs regardless of gender or cancer

**Figure 3:**
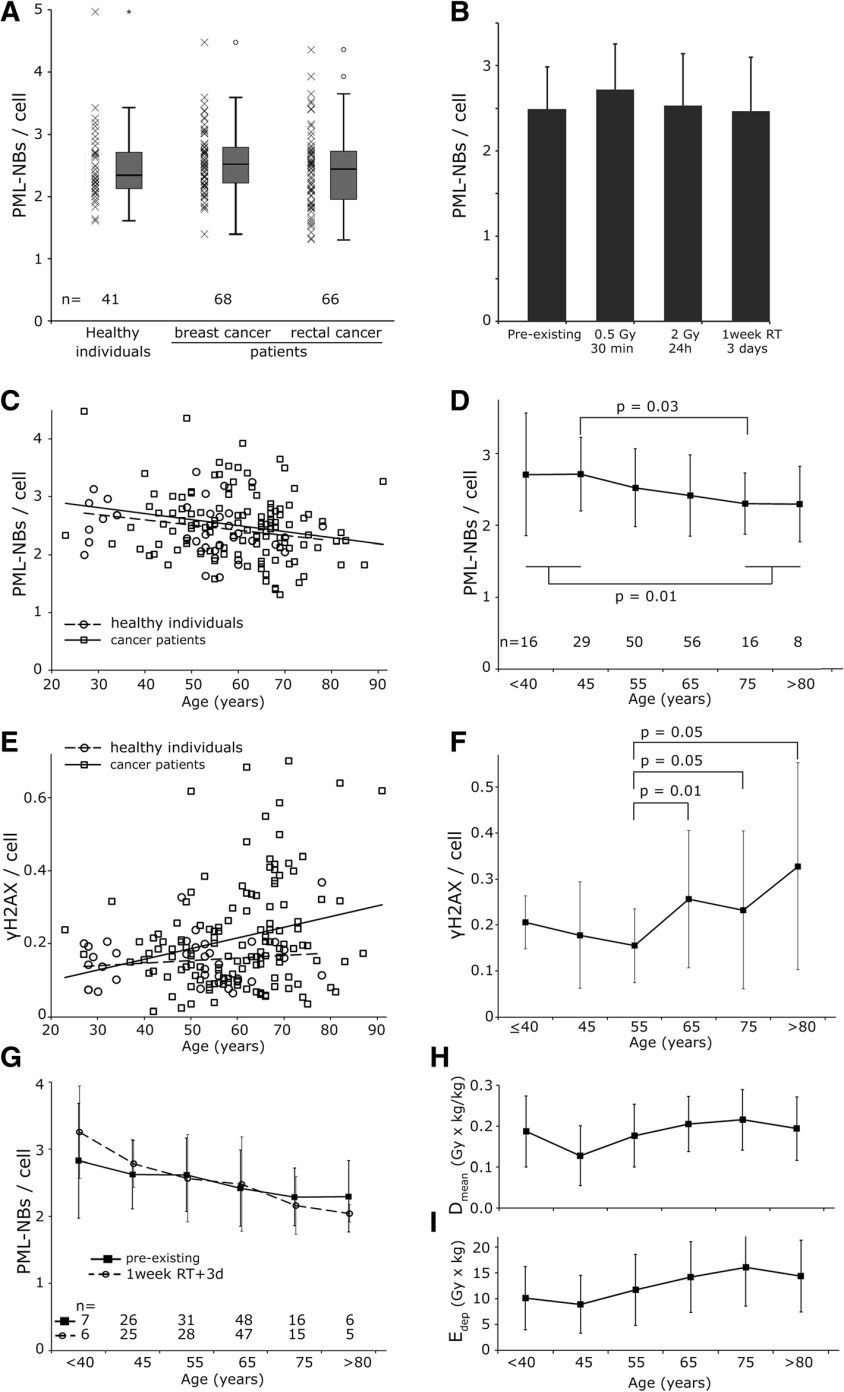
**PML-NBs depending on individuals’ age. (A)** PML-NBs per cell in patients with breast cancer and rectal cancer as well as healthy individuals. **(B)** Mean number of PML-NBs per cell without irradiation, after 0.5 Gy and 30 minutes repair time, after 2 Gy and 24 hours repair time and three days after one week of radiochemotherapy. **(C)** PML-NBs per cell dependent on age. Patients are represented by squares and the healthy individuals by circles. A linear fit was used. Patients are depicted by the solid line and healthy individuals by the dashed line. **(D)** The mean PML-NBs per cell of cancer patients and healthy individuals within groups comprising ten years of age are represented by squares. **(E)** Pre-existing γH2AX foci per cell in dependence of age in healthy individuals (squares) and patients (circles). **(F)** The mean γH2AX foci per cell of patients within groups comprising 10 years of age are represented by squares. There is a significant (p = 0.01) increase in γH2AX per cell between the group comprising 50 to 60 year-old study participants and the group including 60 to 70-year-old individuals. **(G)** Patients PML-NBs per cell divided into age groups comprising ten years of age. The solid line depicts the pre-existing PML-NBs per cell and the dashed line PML-NBs three days after one week of radiochemotherapy. **(H)** D_mean_ and **(I)** E_dep_ in dependence of age comprised in ten-year periods. Error bars depict the standard deviation of the mean.

We used peripheral blood monocytes for the testing because they can be taken easily from a large number of individuals. Per individual 1600 cells in average were analyzed. Samples were divided in equal parts for the (I.) pre-existing PML-NB frequency, (II.) the initial PML foci count after 0.5 Gy and 30 min, (III.) after a 24 h recovery time with 2 Gy and (IV.) after an *in vivo* irradiation. No difference was seen in the existing number of PML-NBs per cell between the healthy individuals group, the patients with BC and the patients with RC (Figure 3A). Within these groups large interindividual differences could be detected.

### Number of PML-NBs without and after various radiation intensities

To determine if radiation induces PML-NBs, we used varied radiation doses. There was no significant difference found between the incidence of existing PML-NBs per cell and the number counted after irradiation with 0.5 Gy and 30 minutes repair time. The incidence of PML-NBs after irradiation with 2 Gy and 24 hours repair time and the number of PML-NBs after 1 week of radiotherapy and 3 days repair time also do not differ significantly from any other experimental approach (Figure 3B).

### PML-NBs per cell decrease with donor age

To determine whether age affects the incidence of PML-NBs, we looked at the nuclei of PBMC from 175 donors aged 22 to 92 years. The mean number of PML-NBs per cell decreases linearly with increasing age at an equivalent level in the control population (−0.0091 PML-NBs per year) and the patient groups (−0.0100 PML-NBs per year) (Figure 3C). The healthy participants and patients were grouped together due to their similar age dependency. Sub-groups comprising ten years of age were formed. The number of PML foci per cell declined significantly (p = 0.03) between the sub-group of 70 to 80 year old individuals compared to the sub-group of 40 to 50 year old individuals and the individuals older than 70 years had statistical significant lower PML-NBs than the study group younger than 50 years (p = 0.01) (Figure 3D).

### γH2AX foci per cell increase with donor age

The linking between PML-NBs and the DNA-damage repair suggests that the decreasing number of PML-NBs leads to an increasing number of γH2AX foci. To investigate the effect of age on the number of γH2AX foci, we used PBMC from the same 175 donors. A growing number of γH2AX foci with increasing age of the patient could be seen (0.0029 γH2AX foci per year). Similarly, the healthy individuals group showed a slight increase as well (0.0007 γH2AX foci per year) (Figure 3E). In the sub-group of 50 to 60 year old patients the number of γH2AX foci per cell nucleus was significantly (p = 0.01) lower than in the sub-group of 60 to 70 year old patients (Figure 3F).

### PML-NBs per cell increase in young and decrease in older individuals after RT

At the age of 40 years and younger the cancer patients’ number of PML-NBs per cell increased after *in vivo* irradiation compared to the pre-existing PML foci (p = 0.15). PBMC from cancer patients aged 70 years and older were found to have higher rates of the pre-existing PML-NBs per cell. After *in vivo* irradiation the number of PML-NBs tended to decrease (p = 0.19). However this variation was not significant. In total, the number of PML-NBs decreased with the donor’s age for the pre-existing PML-NBs as for the values after *in vivo* irradiation (Figure 3G). The number of PML-NBs per cell after *in vitro* irradiation declined significantly (p < 0.001) between the sub-groups of 70 to 80 year old individuals compared to the sub-group of 40 to 50 year old individuals. These effects of *in vivo* irradiation cannot be explained by possible different doses exposed to the groups of donors with different age. Using D_mean_ and E_dep_ to quantify the doses received in Figure 3H, I, it clearly shows that for the 40 to over 80 year old patients the doses are slightly increasing rather than decreasing with age.

## Discussion

One of the major causes of cell aging seems to be impaired repair of DNA damage as a result of changes in cellular stress response which lead to an accumulation of DNA damage [11]. However, there must be several factors leading to dysfunctional DNA repair in aged cells. We were interested in PML because of its involvement in DNA damage repair and stress response. Our major findings are: (i) PML-NBs decrease in PBMC with increasing age of individuals. At the same time the cellular stress increases, which was proved by an accumulation of γH2AX foci in dependence of age. (ii) PML-NBs arise in individuals younger than 50 years after exposure to *in vivo* ionizing radiation. As opposed to this, individuals over 70 years of age show a decrease in the number of PML-NBs as a response to *in vivo* ionizing radiation. There seems to be an impaired PML-NBs stress response in the aged cell. To our knowledge, so far no clinical data exist which depict the age related PML-NBs induction and a limited PML-NB stress response in older individuals.

The impaired stress response in aged cells might be related to the accumulation of DNA damage [12]. The DNA repair is related to PML-NBs in several ways [1]. Gamma-irradiation results in the recruitment of p53 to PML-NBs as one aspect of PML-NBs being involved in DNA repair through several repair proteins [6]. PML-NBs are reported to facilitate the homology-directed repair by interacting with DNA repair proteins such as Rad51 or BML. It makes DSB repair more efficient by facilitating to localize and stabilize Rad51. It is suggested that PML-NBs are involved in processing double-strand breaks, generating ssDNA tails, which are essential for the assembly of DNA repair protein complexes [2]. PML-NBs may have the ability to act as sensors of cellular stress [6]. Although it might be that the accumulation of γH2AX foci is in part a result of the decreased and impaired stress response of the PML-NBs. Our data show that there is a relation between the aging of the cell and the decrease of PML-NBs and it might be related to the change in stress response.

We compared the pre-existing number of PML-NBs per cell in patients suffering from cancer and healthy controls. There was no significant difference in the number of PML-NBs between both groups. Therefore, the decline in the number of PML-NBs seems not to be related with the presence of cancer. Having compared the PML-NBs level in individuals at different ages, we made the observation that increased levels of γH2AX foci coincide with a decrease of PML-NBs in aged cells.

An additional support of the thesis that there seems to be an impaired PML-NBs regulation in dependence of age is that in patients younger than 50 years there was a trend to an increase of PML-NBs after a *in vivo* irradiation. The irradiation consisted of 1.8 Gy daily, five times for one week and three additional days. Quite in contrast to it the individuals between 50 and 70 years old had no change in the number of PML-NBs. Patients older than 70 years even had a reduced number of PML NBs after *in vivo* irradiation.

The PML-NB increase is caused by transcriptional upregulation [13] or fission [3], which results in clear and stable foci, which were made visible by immunostaining. The equally bright PML foci are easy to quantify by high throughput analyses. There is a clear difference of the PML-NBs number between lymphocytes and fibroblasts. Lymphocytes have about 2.5 PML-NBs per cell, while fibroblasts have about 10 PML-NBs per cell. In both cell types PML-NBs rapidly increase in the first three hours after exposure to ionizing radiation. PML-NBs in the lymphocytes decrease very slowly thereafter. In contrary in fibroblasts there is long lasting increase of PML-NBs after stress induction. It might be of higher priority to study the more prominent effect in fibroblasts. However, human PBMC were used for the testing. The advantage of PBMC is that all cells are in G0/G1 phase of cell cycle and so there are no problems arising from the variation of PML-NBs number depending on cell cycle. The disadvantage of PBMC is the low number of PML-NBs and the cells spheroid shape and the resulting problems to count the foci in different planes of the cell. However this can be overcome by acquiring multiple planes by the microscope and fuse it to a single image. Additionally it would have been difficult to have skin biopsies of such a large number of donors and it would not have been possible to yield skin biopsies after one week of RCT. It could be assumed that the presence and alteration of PML-NBs is similar in all cells of the body.

It could be hypothesized that PML monitors a basic aging process and not the effects of a disease [14]. To prove this, we showed that cancer patients have no significant difference in the number of PML-NBs compared to healthy individuals. Since the accumulation of DNA damage is one of the main characteristics of aging [15], it can be assumed that aging might be in some way involved in the DNA repair process. The roles of PML as a sensor of cellular stress and a mediator of DNA damage response is strongly implied by a series of observations made in past studies. PML-NBs were found to be sensitive detectors of cellular stress [3]. Between PML-NBs and DNA repair links have been made, e.g. PML is supposed to have a role in coordination of DSB processing [1]. To support this hypothesis it might be useful to study PML-NBs in animal aging models in the future [16,17].

## Conclusion

The results of our study were generated from a significant number of donors and provide the compelling evidence that there is a dysfunctional stress response in aged individuals. The loss of PML-NBs in number and their impaired stress response seems to be a pathological reaction caused by cell aging. It can be hypothesized that the accumulation of cell damage with increasing age is at least in part caused by an impaired stress response in the aged cell.

## Competing interests

The authors declare that they have no competing interests.

## Authors’ contributions

The experimental work and analysis was carried out by BW, MS, MB, MS. BW and LD wrote the manuscript. LD and RF designed the study. All authors read and approved the final manuscript.

## Pre-publication history

The pre-publication history for this paper can be accessed here:

http://www.biomedcentral.com/1471-2318/14/42/prepub
